# The role of agricultural mechanization services in reducing pesticide input: promoting sustainable agriculture and public health

**DOI:** 10.3389/fpubh.2023.1242346

**Published:** 2023-10-03

**Authors:** Xingchi Li, Mande Zhu

**Affiliations:** ^1^School of Economics, Guizhou University, Guiyang, China; ^2^Business School, Guizhou Education University, Guiyang, China

**Keywords:** public health, agricultural mechanization services, pesticide input, endogenous switching regression, soil pollution

## Abstract

An increasing amount of pesticide input is posing a serious threat to the environment and public health. However, the quantitative analysis of the impact of agricultural mechanization services (AMS) on reducing pesticide input is not yet clear. In this study, the impact of AMS on pesticide input was estimated by using the Chinese Family Database and the endogenous switching regression model. Subsequently, testing the robustness of the model using the substitution variable method. The impact of AMS on different types of pesticides and the influence of farmers' choices of AMS on pesticide input under different terrain conditions and farm sizes were analyzed as well. The results demonstrate that AMS has a significant and negative impact on pesticide input, reducing pesticide expenditures by 56.08% for farmers who adopt AMS. For farmers who do not adopt AMS, adopting such services is assumed to reduce pesticide input by 14.97%. AMS can also reduce the use of insecticides and herbicides by over 54%. Compared to mountainous and hilly areas, AMS in plain areas increase the effectiveness of pesticide input reduction by 30.40%. Furthermore, small-scale farmers who adopt AMS can increase pesticide input reduction by 90 yuan/mu compared with large-scale farmers. Therefore, promoting the development of socialized AMS, strengthening AMS in mountainous and hilly areas, and expanding the scale of operation for small-scale farmers can help improve the effectiveness of pesticide input reduction. The results of this study can inform the development of strategies to reduce chemical pesticide use in agricultural soil.

## 1. Introduction

Chemical pesticides are widely used in agricultural production to control pests and weeds and increase crop productivity (1, 2). However, with the development of modern agriculture, the use of chemical pesticides has increased continuously, posing serious threats to the environment and human health (3, 4). Approximately 2 million tons of pesticides are used worldwide each year, with China being the major contributing country, followed by the United States and Argentina (5). Approximately 64% of agricultural land worldwide is at risk of environmental pollution caused by pesticide use (6). Addressing food security issues based on pesticides requires addressing the impact of pesticides on human health and the environment simultaneously (7). The link between pesticide use and immune suppression, hormonal disruption, cognitive decline, reproductive abnormalities, and cancer is becoming increasingly evident (8). Pesticide use increases the risk of human cancer, and in the southern and southeastern regions of Brazil, pesticide levels and colon cancer cases have almost doubled in 14 years (9). Therefore, effectively reducing pesticide inputs and mitigating the negative impact on the environment and human health has become a hot topic of concern while addressing food security by increasing agricultural productivity.

Agricultural machinery contributes to enhancing agricultural productivity and reducing the unit cost of agricultural production (10). The provision of professional agricultural mechanization services (AMS) by agricultural cooperatives and agribusinesses plays an important role in promoting the utilization of agricultural machinery in China, considering the high cost of agricultural machinery inputs. Developing and training providers of AMS is an important approach to promoting small farmers' access to such services (11). Productivity gains from mechanization services in China are more pronounced among medium-scale farmers compared to small and large-scale farmers (12). Moreover, the impact of farmers' adoption of AMS on land leasing is weakened by the topography of non-flat rural villages (13). As labor mobility promotes the adoption of AMS, the impact of AMS on agricultural efficiency is significant and positive (14). A 1% increase in the ratio of scale of farming is associated with a 0.017% decrease in the use of pesticides per unit (15). Thus, AMS has a significant impact on the allocation of agricultural factors, which may vary depending on factors such as farm size and topography.

In general, the impact of mechanization on pesticide input in agriculture mainly includes the following three aspects: (a) mechanization promotes precise pesticide spraying, which is beneficial for reducing pesticide input. (b) Mechanized cultivation and weed control in agriculture can reduce farmers' reliance on chemical pesticides, thereby decreasing pesticide input in production. (c) The adoption of agricultural mechanization may help prevent and control crop pests and weeds, thus reducing the need for pesticide input. Agricultural mechanization can avoid losses and waste in pesticide input through precise measurement, effectively improve agricultural productivity and reduce unit production costs (16). The use of agricultural drones for spraying can effectively reduce the amount of fertilizers, pesticides, and water needed in farming (17). The use of agricultural machinery had a significant negative effect on pesticide expenditures based on a survey of 493 corn growers in China (18). However, a quantitative analysis of the impact of AMS on pesticide input under different farm sizes and topographical conditions in China is still lacking.

Farmers' choice of socialized AMS may be influenced by observed and unobserved factors related to pesticide expenditures, leading to sample selection bias and endogeneity issues. Furthermore, AMS may have differential effects on the inputs of different types of pesticides, including insecticides and herbicides. Differences in the rate of AMS utilization across varying terrain conditions may contribute to disparities in pesticide input reduction. Due to the higher ownership rate of agricultural machinery among large-scale farmers, the impact of small-scale farmers' adoption of AMS on pesticide input remains unclear.

Based on the theoretical and research foundation outlined above, this study proposes the following hypotheses:

Hypothesis 1: AMS can effectively reduce pesticide input.
Hypothesis 2: The reduction in pesticide input achieved by AMS is higher in plain regions compared to mountainous and hilly regions.
Hypothesis 3: Large-scale farmers experience a higher reduction in pesticide input compared to small-scale farmers due to the scaling effects of AMS.

This study aims to investigate the influence of AMS on pesticide input, to provide guidance for the government to reduce pesticide input. Specifically, this research utilizes the Chinese Family Database (CFD) and employs the endogenous switching regression (ESR) model to address the endogeneity issue in the relationship between AMS and pesticide input. The study explores the essential factors determining farmers' decisions on AMS and pesticide input, and identifies the differences in the impact of AMS on pesticide input about different types of pesticides, terrain conditions, and farm sizes. The results of this study contribute to a better understanding of the effect of AMS on pesticide input and the environment, and offer scientific support for the sustainable agriculture and public health.

## 2. Methodology and data

### 2.1. Data source

**Figure 1:**
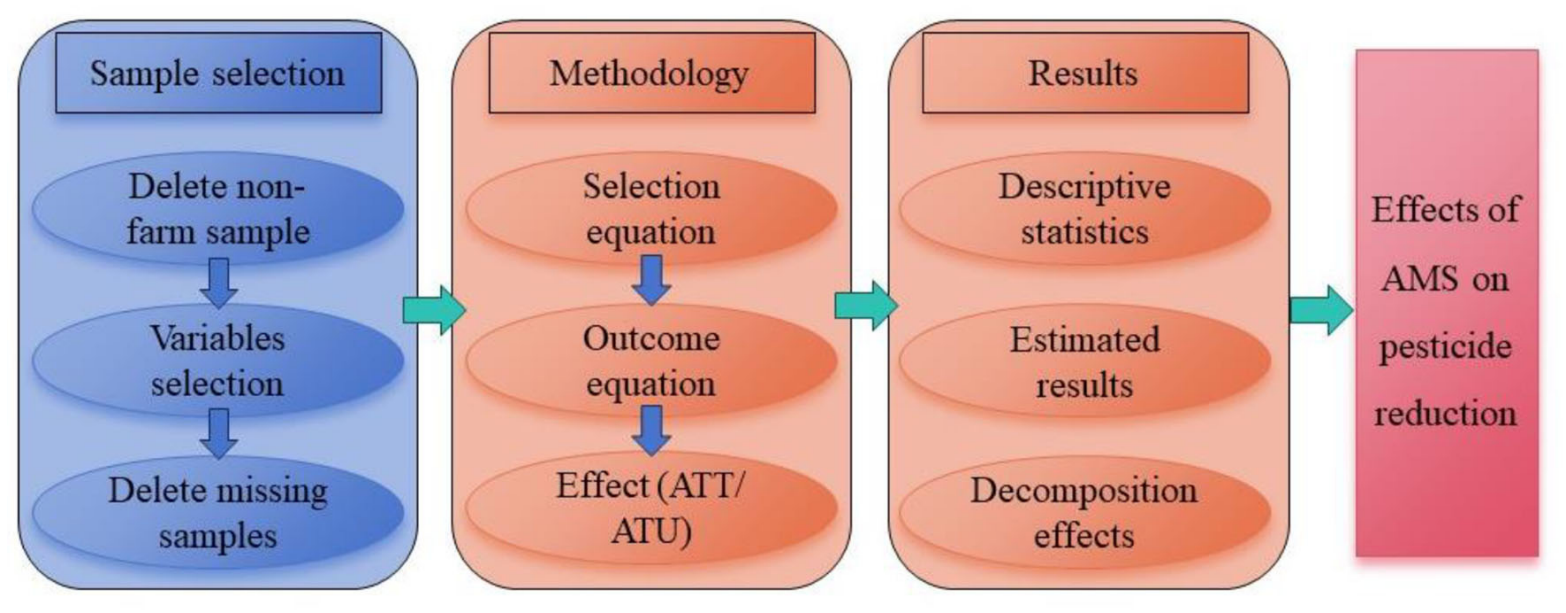
Sample selection and research process.

The data of this study is based on Chinese Family Database (CFD) of Zhejiang University collected in 2017. This database was chosen because it represents the most recent publicly available data, which has been impacted by the COVID-19 pandemic. The survey data includes information on Chinese rural households such as basic information, employment, income and expenditure, agricultural production, land use and transfer, social security, and education. On the basis of CFD, the data were cleaned to obtain the research sample in the following ways (Figure 1): firstly, the samples that were not engaged in agricultural production operations were deleted. Subsequently, selected the variables needed for the study. Finally, the samples with missing information or “don't know” answers were deleted. A total of 11,942 samples were obtained from 482 villages in 29 provinces in China. Among them, the samples that chose and did not choose AMS for agricultural production were 7538 and 4406, respectively.

### 2.2. Variable selection

The selection of dependent variables in this study mainly focuses on farmers' pesticide reduction behavior. As shown in Table 1, four indicators are used to reflect the pesticide expenditure and usage of farmers in their production and operation [e.g., per pesticide expenditure, per insecticide expenditure, per herbicide expenditure, and per pesticide use (15 mu = 1 hectare)]. The per pesticide expenditure represents the ratio of annual pesticide use expenditure to farmland area in the sample. The per insecticide expenditure and per herbicide expenditure represent the ratio of annual insecticide expenditure and herbicide expenditure to farmland area, respectively. Given the price differences of pesticides in different regions, the per pesticide use is used to measure the intensity of pesticide use in the sample. The per pesticide use is calculated based on the pesticide price index and the average pesticide price in different provinces in China Rural Statistical Yearbook.

**Table 1:** Definition of variables and descriptive statistics.

**Variables**	**Definition**	**Mean**	**Standard deviation**
**Explained variables**			
Pesticide expenditure	Pesticide expenditure per unit of cropland area (yuan/mu)	180.327	672.475
Insecticide expenditure	Insecticide expenditure per unit of cropland area (yuan/mu)	136.120	598.004
Herbicide expenditure	Herbicide expenditure per unit of cropland area (yuan/mu)	44.207	133.735
Pesticide use	Pesticide use per unit of cropland area (kg/mu)	7.229	27.018
**Core explanatory variable**			
Agricultural mechanization services	Whether to rent agricultural machinery: Yes = 1, No = 0	0.631	0.483
**Control variables**			
Age	The age in 2017	46.986	18.040
Gender	Male =1, female =0	0.515	0.500
Educational level	High school education or above: Yes = 1, No = 0	0.187	0.390
Health status	1 = Very good, 2 = Good, 3 = Fair, 4 = Poor, 5 = Very poor	2.723	1.093
Agricultural labor force	The average number of laborers during busy farming and non-busy farming seasons	1.877	0.767
Household income	Disposable household income in 2017 (10000 yuan)	5.304	6.502
Cultivated land scale	Cultivated land area (mu)	11.565	22.151
Number of land plots	Number of land plots	6.239	6.742
Agricultural subsidies	1=Received agricultural subsidies, 0=No	0.765	0.424
Self-owned agricultural machinery	1 = Yes, 0 = No	0.520	0.500
Hired laborers	1 = Yes, 0 = No	0.148	0.355
Purchasing agricultural supplies through e-commerce platforms	1 = Yes, 0 = No	0.188	0.391
Distance from village	Distance in kilometers from the nearest township to the village (km)	6.997	6.341
Per capita income of village	Per capita disposable income of rural residents in the village (10,000 yuan)	0.709	0.573
Economic crops ratio in the village.	The economic crop planting area ratio in the village (as a percentage)	33.885	31.350
**Instrumental variables**			
Village agricultural mechanization services	The proportion of agricultural mechanization service samples in the village (excluding this sample)	0.606	0.322

The core explanatory variable is whether farmers adopt socialized AMS, which is measured by whether the sample rents agricultural mechanization or not. The adoption of socialized AMS is coded as 1, otherwise it is coded as 0. According to the sample in the database, if the expenditure on renting agricultural mechanization is 0, it means that socialized AMS are not chosen. Conversely, if the expenditure on renting agricultural mechanization is greater than 0, it means that socialized AMS are chosen.

To illustrate the potential impact of other factors on farmers' pesticide reduction behavior, this study considers personal characteristics, household characteristics, and village characteristics as control variables. In terms of personal characteristics, the factors such as age, gender, education level, and health status are considered. In terms of household characteristics, the factors such as the number of family laborers, family income, farmland scale, number of farmland plots, agricultural subsidies, agricultural mechanization, hiring laborers, and purchase agricultural inputs through e-commerce platforms are adopted. In terms of village characteristics, the factors such as village geographical location, village economic development level, and the proportion of economic crops in the village are considered. By controlling these variables, the relationship between farmers' pesticide reduction behavior and their personal, household, and village characteristics can be better revealed. Thus, eliminate the influence of other factors on research results, and improve the reliability and accuracy of the research.

In this study, the village AMS rate is used as an instrumental variable to address endogeneity issues. There is a mutual influence between farmers' mechanization use and pesticide use. If we directly use socialized AMS as the explanatory variable, it may lead to endogeneity issues. The village AMS rate can reflect the popularity of mechanization use in the village, but does not directly affect farmers' pesticide use. It can be used as an instrumental variable to eliminate endogeneity issues. By using instrumental variables, we can more accurately estimate the response of farmers' pesticide reduction behavior to mechanization use.

### 2.3. Methodology

Generally, farmers' choice of AMS is a self-selection problem based on observed and unobserved factors. Hence, the self-selection of farmers results in endogeneity bias in the estimated outcomes of the model. In this study, it is assumed that farmers are risk neutral and they consider the expected benefits of choosing AMS ($D_{c}^{*}$) and the expected benefits of non-choosing AMS ($D_{n}^{*}$). The difference between the expected benefits of choosing and non-choosing AMS is defined as $D_{i}^{*}$ (i.e., $D_{i}^{*}=D_{c}^{*}-D_{n}^{*}$). If $D_{i}^{*}$ > 0, the farmer chooses AMS. $D_{i}^{*}$ cannot be observed directly due to its subjective nature, but it can be expressed in a latent variable model as a series of equations with observable variables. The factors influencing the adoption of AMS by farmers can be represented by the following selection equation:


(1) $$D_{i}^{*}=\alpha Z_{i}+\mu_{i},\;D_{i}=\left\{ \begin{array}{l} 1\;if\;D_{i}^{*}>0\\ 0\text{ otherwise} \end{array}\right.$$

where *D*_*i*_ is a binary variable, *Z*_*i*_ is a vector of factors influencing the choice of AMS (e.g., characteristics of households and pesticide inputs), α is the unknown coefficient of the corresponding variable, and μ_*i*_ is an error that follows a normally distributed with zero means.

The two outcome equations are expressed as follows under choosing and non- choosing of the AMS by farmer *i*.


(2) $$\left\{ \begin{array}{l} Y_{ci}=\beta_{ci}X_{i}+\varepsilon_{ci}\;if\;\;D_{i}=1\\ Y_{ni}=\beta_{ni}X_{i}+\varepsilon_{ni}\;if\;\;D_{i}=0 \end{array}\right.$$

where *Y*_*ci*_ and *Y*_*ni*_ are the outcome variables (i.e., pesticide expenditure and pesticide use), *X*_*i*_ is the vector of explanatory variables that may impact pesticide input, β_*ci*_ and β_*ni*_ are the unknown coefficient of the corresponding variable, ε_*ci*_ and ε_*ni*_ are the error terms associated with the outcome variables.

The three error terms μ_*i*_, ε_*ci*_ and ε_*ni*_ in Equations (1) and (2) are assumed to have a trivariate normal distribution with mean vector zero and covariance matrix (19).


(3) $$\Omega =\left[\begin{array}{ccc} \sigma_{u}^{2} & \sigma_{cu} & \sigma_{nn}\\ \sigma_{cu} & \sigma_{c}^{2} & \cdot \\ \sigma_{nu} & \cdot & \sigma_{n}^{2} \end{array}\right]$$

where $\sigma_{u}^{2}$ is a variance of the error term in the selection equation (1), $\sigma_{c}^{2}$ and $\sigma_{n}^{2}$ n are the variances of the error terms in the continuous equation (2), σ_*cu*_ is a covariance of μ_*i*_ and ε_*ci*_, σ_*nu*_ is a covariance of μ_*i*_ and ε_*ni*_. The covariance between ε_*ci*_ and ε_*ni*_ is not defined as *Y*_*ci*_ and is never observed simultaneously. The expected values of the error terms ε_*ci*_ and ε_*ni*_ conditional on the sample selection are non-zero and are defined as:


(4) $$\begin{array}{l} E\left(\varepsilon_{ci}|D_{i}=1\right)=\sigma_{cu}\frac{\varphi \left(\alpha Z_{i}\right)}{\Phi \left(\alpha Z_{i}\right)}=\sigma_{cu}\lambda_{ci} \end{array}$$

(5) $$\begin{array}{l} E\left(\varepsilon_{ni}|D_{i}=0\right)=\sigma_{nu}\frac{\varphi \left(\alpha Z_{i}\right)}{1-\Phi \left(\alpha Z_{i}\right)}=\sigma_{nu}\lambda_{ni} \end{array}$$

where φ(·) and Φ(·) are the probability density function and cumulative density function of standard normal distribution, respectively.

The coefficients from the ESR model can be employed to calculate the average treatment effect on the treated (ATT). The observed and unobserved counterfactual outcomes for choosing AMS are presented as follows (20).


(6) $$\begin{array}{l} E\left(Y_{ci}|D_{i}=1\right)=X_{i}\beta_{ci}+\sigma_{cu}\lambda_{ci} \end{array}$$

(7) $$\begin{array}{l} E\left(Y_{ni}|D_{i}=1\right)=X_{i}\beta_{cn}+\sigma_{nu}\lambda_{ci} \end{array}$$

The unbiased average treatment effect on the treated (ATT) can be obtained by the expected outcomes of Equations (6) and (7).


(8) $$\begin{array}{l} ATT\;=\;E(Y_{ci}|D_{i}=1)-E(Y_{ni}|D_{i}=1)\\ \;=(\beta_{ni}-\beta_{ci})X_{i}+(\sigma_{cu}-\sigma_{nu})\lambda_{ci} \end{array}$$

The average treatment effect on untreated (ATU) can be expressed as follows:


(9) $$\begin{array}{l} ATU\;=\;E(Y_{ci}|D_{i}=0)-E(Y_{ni}|D_{i}=0)\\ \;=X_{i}(\beta_{ci}-\beta_{ni})+\lambda_{ni}(\sigma_{cu}-\sigma_{nu}) \end{array}$$

## 3. Results and discussions

### 3.1. Descriptive statistics

**Figure 2:**
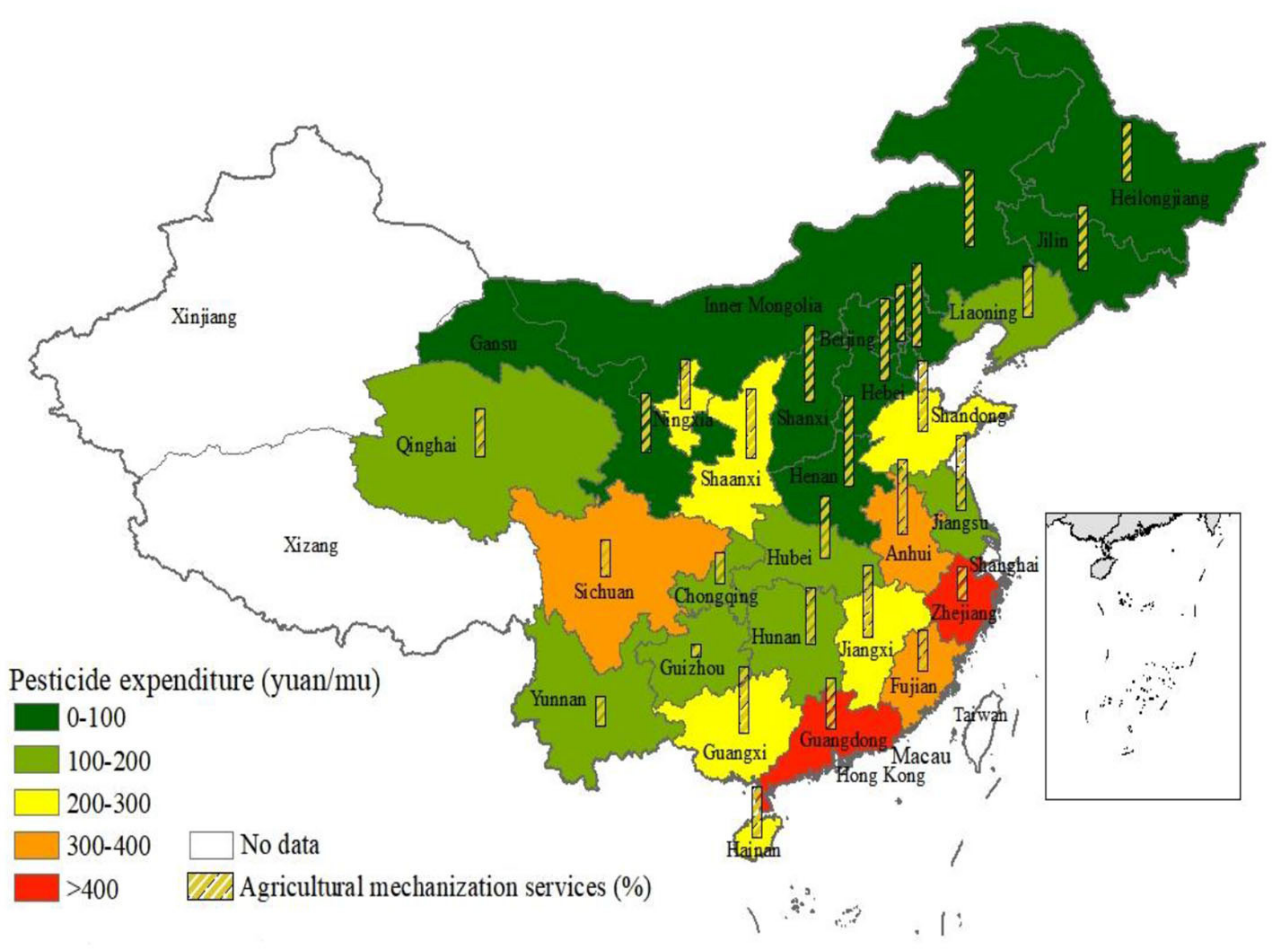
Pesticide expenditure and AMS of sample provinces.

The results of the descriptive statistical analysis showed that the pesticide expenditure varied significantly among households, with an average expenditure of 180.327 yuan/mu (Table 1). The analysis revealed that the pesticide expenditure for insecticides was 91.913 yuan/mu higher than for herbicides. Additionally, the pesticide use was found to be 7.229 kg/mu on average. According to the average pesticide expenditure in the sample provinces, Zhejiang and Guangdong provinces have the highest pesticide expenditure, while the provinces with the least pesticide expenditure are mainly located in the plain areas and major grain-producing areas (Figure 2). Overall, in provinces where the pesticide expenditure is less than 100 yuan/mu, the agricultural machinery service rate is over 60% on average. Among them, the agricultural machinery service rate in Henan province is as high as 95%. In contrast, in other provinces with higher pesticide expenditure, the AMS rate is relatively low. Similarly, the results of Quan and Doluschitz indicate that the maize farmers in the northeast and northern regions of China are more likely to use agricultural machinery than in other areas (21).

The statistical results indicate that 63% of the sampled households adopt socialized AMS, while 52% of the samples own agricultural machinery. The average age of the samples is relatively high, with a mean of around 47 years old. Less than 20% of the samples received education at or above the high school level. The farmland scale is relatively small and fragmented, with an average farmland area of 11.565 mu and an average number of land plots exceeding 6. More than 76% of the sampled households received agricultural subsidies, while less than 20% hired agricultural laborers or were willing to purchase agricultural inputs through e-commerce platforms.

The analysis also showed that households in mountainous and hilly areas tended to use more pesticides than those in plains areas, with an average pesticide expenditure of 209.781 yuan/mu and 140.158 yuan/mu, respectively (Table 2). According to the definition by the World Bank, small-scale farmers are those who cultivate less than 30 mu of land (22). The analysis revealed that large-scale farmers tended to use fewer pesticides than small-scale farmers, with an average pesticide expenditure of 63.417 yuan/mu and 190.063 yuan/mu, respectively. Similarly, Qiu et al.'s analysis of household data in Henan, China, indicates that large-scale farmers are more willing to purchase their own agricultural machinery rather than Adoption of AMS (12). It should be noted that the socialized AMS rate and the self-owned mechanization rate in the plain areas are higher than those in the mountainous and hilly areas, with a difference of 23% and 7%, respectively. However, large-scale farmers have a lower socialized AMS rate than small-scale farmers, with an average reduction of 7%. This may be related to the fact that large-scale farmers have a higher proportion of self-owned mechanization, which accounts for as much as 89%.

**Table 2:** Mean values of AMS and pesticide use under different terrains and farmland scales.

**Variables**	**Nationwide**	**Terrain**		**Cultivated land scale (mu)**	
		**Plain**	**Mountains and hills**	<**30 mu**	≥**30 mu**
Pesticide expenditure per mu (yuan/mu)	180.327	140.158	209.781	190.063	63.417
Insecticide expenditure per mu (yuan/mu)	136.120	103.173	160.278	143.896	42.747
Herbicide expenditure per mu (yuan/mu)	44.207	36.985	49.503	46.167	20.670
Pesticide use per mu (kg/mu)	7.229	5.652	8.385	7.619	2.546
Agricultural mechanization services	0.631	0.763	0.535	0.636	0.566
Self-owned agricultural machinery	0.520	0.558	0.493	0.490	0.886
Samples	11,942	5,052	6,890	1,1024	918

### 3.2. Estimation results of the ESR model

#### 3.2.1. Determinants of choosing AMS

In this study, the full information maximum likelihood (FIML) method to jointly estimate the selection equation and outcome equation was used to obtain parameter estimates. The estimation results are presented in Table 3. The results of the parameter estimates for the selection equation indicate that the personal, household, and production characteristics of farmers have a significant impact on their decision to choose AMS. The results show that the education level of farmers has a significant and positive coefficient, indicating that farmers with a high school education or above are more inclined to adopt AMS. Furthermore, the results indicate that the coefficient of household income is significant and positive, suggesting that farmers with higher household incomes are more likely to choose AMS. Ma et al. found that the income effect of off-farm work stimulates agricultural production by increasing inputs that enhance productivity (23). The provision of agricultural subsidies has the potential to encourage farmers to adopt AMS. This can be attributed to the fact that such subsidies reduce the cost of agricultural services, thereby making it easier and more affordable for farmers to access these services. Similarly, Salehi and Rasoulaizar pointed out that factors affecting the effectiveness of agricultural machinery cooperatives include economic, managerial, social, policy, and educational factors (22).

**Table 3:** Analysis of factors affecting the adoption of AMS and pesticide inputs.

**Variables**	**Selection equation**	**Pesticide input**	
		**Adoption of AMS**	**Non-adoption of AMS**
Age	0.001 (0.001)	−0.598^*^ (0.326)	−0.712 (0.866)
Gender	−0.034 (0.029)	−6.408 (10.327)	2.758 (27.473)
Educational level	0.093^**^ (0.041)	−16.932 (13.907)	123.940^***^ (39.786)
Health status	−0.003 (0.015)	−5.931 (5.239)	15.928 (14.053)
Agricultural labor force	0.002 (0.019)	−0.785 (6.932)	0.252 (17.855)
Household income	0.013^***^ (0.003)	0.0148 (0.773)	2.279 (2.527)
Cultivated land scale	−0.001 (0.001)	−3.314^***^ (0.305)	−1.397^***^ (0.529)
Number of land plots	−0.002 (0.002)	8.586^***^ (1.015)	−5.827^***^ (1.673)
Agricultural subsidies	0.159^***^ (0.035)	−98.914^***^ (13.534)	−129.257^***^ (30.413)
Self-owned agricultural machinery	−0.636^***^ (0.031)	50.531^***^ (11.319)	17.371 (31.669)
Hired laborers	−0.193^***^ (0.042)	−169.058^***^ (14.940)	−64.734 (40.405)
Purchasing agricultural supplies through e-commerce platforms	0.118^***^ (0.037)	−25.308^*^ (13.594)	−90.011^***^ (34.374)
Distance from village	−0.002 (0.002)	−0.241 (0.957)	−5.547^***^ (1.881)
Per capita income of village	−0.029 (0.030)	57.059^***^ (11.736)	76.279^***^ (18.953)
Economic crops ratio in the village.	−0.001 (0.001)	1.037^***^ (0.173)	3.175^***^ (0.441)
Instrumental variables	3.177^***^ (0.055)		
Constant term	−0.944^***^ (0.123)	496.884^***^ (42.663)	403.876^***^ (112.290)
Lnσ_cu_		6.099^***^ (0.008)	
ρ_cu_		−0.032 (0.021)	
Lnσ_nu_			6.811^***^ (0.011)
ρ_nu_			0.114^***^ (0.023)
LR test of indep. Eqns.	18.78^***^		
Log likelihood	−97,633.471		
Observed value	11,942		

^***, **, *^ indicates significance at the 1%, 5%, 10% level, respectively. Standard errors are reported in parentheses.

It should be noted that purchasing agricultural inputs through e-commerce platforms has a significant positive coefficient. In other words, farmers who purchase agricultural inputs through e-commerce platforms are more likely to choose AMS. This may be because these farmers are more willing to adopt advanced agricultural technologies and have easier access to information on AMS. Farmers hiring laborers have a significant negative impact on the adoption of AMS, suggesting that hiring laborers may decrease the probability of selecting these services by farmers. Moreover, farm ownership of agricultural machinery has a significant negative effect. Specifically, farmers who already own agricultural machinery are less inclined to purchase AMS compared to those who do not own any agricultural machinery. Qiu et al. indicated that large farms are more willing to invest in self-owned agricultural machinery rather than adopting AMS because large farms can provide specialized AMS to other farms, which can shorten the break-even period (12).

#### 3.2.2. Factors influencing pesticide inputs

In this study, a likelihood ratio test was employed to examine the joint independence between the selection equation and the outcome equation. As presented in Table 3, the chi-square statistic was 18.78, which was significant at the 1% level, indicating that the selection and outcome equations were not independent and required simultaneous estimation. This finding also confirms the suitability of the ESR model. Table 3 presents the estimated determinants of pesticide inputs, as reported in the third and fourth columns, which correspond to the use or non-use of AMS. For farmers who use AMS, age, cultivated land size, agricultural subsidies, hired labor, and purchasing agricultural inputs through e-commerce platforms have a significant negative effect on pesticide inputs. In contrast, the number of cultivated plots, ownership of agricultural mechanization, per capita income in the village, and the proportion of cash crops in the village have a significant positive effect on pesticide inputs. Ma and Zheng indicated that smartphone-based information interventions can guide the appropriate use of agricultural chemicals, which helps to reduce the excessive usage of pesticides and fertilizers (24).

For farmers who do not use AMS, cultivated land size, number of cultivated plots, agricultural subsidies, purchasing agricultural inputs through e-commerce platforms, and village distance have a significant negative effect on pesticide inputs. Aubert and Enjolras found that European Union subsidies (second pillar) led to a decrease in pesticide expenditure, excluding crop insurance subsidies (25). In contrast, farmer education level, per capita income in the village, and the proportion of cash crops in the village have a significant positive effect on pesticide inputs. This may be related to risk-averse farmers who are more likely to use more pesticides (26). Similarly, Tan et al. pointed out that education level, policy guidance, and environmental awareness are the main factors that affect pesticide use (27).

#### 3.2.3. The impact of AMS on pesticide inputs

The estimated results of the average treatment effect for the treatment group (ATT) and the average treatment effect for the untreated group (ATU) based on the ESR model are presented in Table 4. The estimated results of ATT suggest that choosing AMS can significantly reduce pesticide inputs by 56.08%. The results of ATU also indicate a reduction effect of AMS on pesticide inputs. Specifically, for farmers who do not use AMS, assuming that they choose to use such services would result in a 14.97% reduction in pesticide inputs. Similarly, Zhang et al. found that choosing to use agricultural machinery can reduce pesticide expenditures by about 59%, while households not using machinery assumed that choosing to use machinery would reduce pesticide expenditures by approximately 33% (18). Thus, Hypothesis 1 has been validated. Moreover, specialized agricultural services have a negative impact on pesticide application intensity (28). Femenia et al. found that pesticide taxes appear to result in a significant reduction in pesticide use but require support for technological advancements to improve low-input cropping practices (29). Thus, the adoption of AMS helps to reduce pesticide inputs, thereby reducing the impact of pesticide exposures in crops on human health.

**Table 4:** The impact of AMS on pesticide expenditure.

**Variables**	**Estimation**	**Mean pesticide expenditure (yuan/mu)**		**Treatment effects**	***t*-value**	**Reduction rate (%)**
		**Adoption of AMS**	**Non-adoption of AMS**			
Pesticide expenditure	ATT	141.854 (1.261)	323.016 (1.802)	−181.162^***^	−82.355	56.08
	ATU	209.346 (2.156)	246.217 (2.992)	−36.870^***^	−9.998	14.97

^***^ indicate significance at the 1% level. Standard errors are reported in parentheses.

### 3.3. Robustness test

To assess the robustness of the results obtained above, the substitution variable method was utilized (30). Subsequently, the explanatory variable of average pesticide expenditure per mu was replaced with average pesticide use per mu, and the model was re-estimated accordingly (Table 5). After replacing the explanatory variable of average pesticide expenditure per mu with average pesticide usage per mu using the substitution variable method, the estimated results in Table 5 remain consistent with those in Table 4. And both ATT and ATU show significant negative effects at the 1% level. This indicates that AMS still has a pesticide reduction effect, suggesting that the influence of AMS on pesticide reduction is robust.

**Table 5:** The impact of AMS on pesticide use.

**Variables**	**Estimation**	**Mean pesticide use (kg/mu)**		**Treatment effects**	**t-value**	**Reduction rate (%)**
		**Adoption of AMS**	**Non-adoption of AMS**			
Pesticide use	ATT	5.696 (0.051)	12.930 (0.072)	−7.234^***^	−82.270	55.95
	ATU	8.408 (0.086)	9.855 (0.119)	−1.447^***^	−9.825	14.68

^***^ indicate significance at the 1% level. Standard errors are reported in parentheses.

### 3.4. Decomposition effects of AMS on pesticide input

#### 3.4.1. Type of pesticide input

To analyze the average treatment effects of AMS on different types of pesticide expenditure, we obtained the ATT and ATU estimates of the impact of AMS on insecticide and herbicide expenditure (Table 6). The ATT estimates indicate that farmers who adopt AMS reduce their insecticide expenditure by up to 57.05%, and their herbicide expenditure by up to 54.64%. The ATU estimates suggest that for non-adopters of AMS, assuming a 16.53% reduction in insecticide expenditure and an 8.53% reduction in herbicide expenditure due to the adoption of AMS. According to previous studies, some insecticides have a negative impact on the normal functioning of the human nervous system, while the herbicide glyphosate is linked to cancer (31). Overall, the pesticide reduction effect of AMS is greater for insecticides than for herbicides when adopted by farmers.

**Table 6:** The impact of AMS on different types of pesticide expenditure.

**Variables**	**Estimation**	**Mean pesticide expenditure (yuan/mu)**		**Treatment effects**	**t-value**	**Reduction rate (%)**
		**Adoption of AMS**	**Non-adoption of AMS**			
Insecticide expenditure	ATT	104.734 (0.991)	243.840 (1.615)	−139.106^***^	−73.416	57.05
	ATU	158.502 (1.699)	189.888 (2.588)	−31.385^***^	−10.137	16.53
Herbicide expenditure	ATT	37.119 (0.277)	81.837 (0.297)	−44.717^***^	−0.011	54.64
	ATU	51.518 (0.468)	56.323 (0.537)	−4.805^***^	−6.747	8.53

^***^ indicate significance at the 1% level. Standard errors are reported in parentheses.

#### 3.4.2. Type of terrain

To investigate the impact of farmers' adoption of AMS on pesticide input under different terrain conditions, we estimated the ATT and ATU results of pesticide expenditure for plain and mountainous and hilly areas, respectively (Table 7). The classification of terrain was based on the criteria provided by the National Bureau of Statistics of China. In plain areas, the pesticide reduction effect of AMS adopted by farmers can reach up to 74.65%. For non-adopters of AMS, assuming adoption could result in a 14.66% reduction in pesticide expenditure in plain areas. For mountainous and hilly areas, the ATT estimates indicate that AMS can reduce pesticide expenditure by 44.25%. The ATU results also reveal a reduction effect of 16.04% on pesticide expenditure due to the adoption of AMS. Xie et al. showed that compared to farmers in plain areas, farmers in mountainous areas had a significant impact on promoting the reduction of pesticide, fertilizer, and plastic film inputs (32). The results suggest that the reduction effect of AMS on pesticide expenditure is generally greater in plain areas than in mountainous and hilly areas. Correspondingly, this validates Hypothesis 2.

**Table 7:** The impact of AMS on pesticide expenditure under different terrain conditions.

**Variables**	**Estimation**	**Mean pesticide expenditure (yuan/mu)**		**Treatment effects**	**t-value**	**Reduction rate (%)**
		**Adoption of AMS**	**Non-adoption of AMS**			
Plain areas	ATT	121.594 (2.068)	479.657 (2.726)	−358.064^***^	−0.001	74.65
	ATU	170.100 (5.027)	199.309 (5.695)	−29.209^***^	−3.846	14.66
Mountainous and hilly areas	ATT	162.960 (1.710)	292.328 (3.014)	−129.368^***^	−37.331	44.25
	ATU	221.337 (2.431)	263.612 (3.990)	−42.275^***^	−9.048	16.04

^***^ indicate significance at the 1% level. Standard errors are reported in parentheses.

#### 3.4.3. Type of cropland scale

The ATT and ATU effects of AMS on pesticide input were estimated separately for small-scale farmers (i.e., the cropland size less than 30 mu) and large-scale farmers, and the results are presented in Table 8. The results from Table 8 show that the treatment effect of AMS on pesticide expenditure for small-scale farmers is −133.122 yuan/mu, while for large-scale farmers, the treatment effect is −42.464 yuan/mu, as estimated by ATT. The ATU results suggest that for non-adopters of AMS, assuming adoption could lead to a reduction of 3.50% and 22.86% in pesticide expenditure for small-scale and large-scale farmers, respectively. Gao et al. showed that the small and scattered farms resulted in increased pesticide use in China (33). Zhang et al. showed that with the increase of farm size, pesticide expenditure decreased by about 3.33 yuan/mu (18). This may be related to the frequent application of pesticides by small-scale farmers to maintain higher productivity (34). It means that large-scale farmers can further reduce the impact of pesticides on public health and the environment through AMS. Therefore, the validation of Hypothesis 3 confirms that the reduction in pesticide input is more pronounced among large-scale farmers implementing AMS compared to small-scale farmers.

**Table 8:** The impact of AMS on pesticide expenditure for different cropland scale farmers.

**Variables**	**Estimation**	**Mean pesticide expenditure (yuan/mu)**		**Treatment effects**	**t-value**	**Reduction rate (%)**
		**Adoption of AMS**	**Non-adoption of AMS**			
Small-scale farmers	ATT	148.269 (1.571)	281.391 (2.510)	−133.122^***^	−44.967	47.31
	ATU	254.187 (2.424)	263.405 (3.888)	−9.218^***^	−2.012	3.50
Large-scale farmers	ATT	55.719 (1.365)	98.183 (1.868)	−42.464^***^	−18.355	43.25
	ATU	56.701 (2.200)	73.503 (2.673)	−16.802^***^	−4.853	22.86

^***^ indicate significance at the 1% level. Standard errors are reported in parentheses.

## 4. Conclusions

The main findings of this study are as follows: (a) AMS has a significant negative impact on pesticide input, reducing pesticide expenditure by 56.08% for adopters of AMS. For non-adopters, assuming the adoption of AMS could lead to a reduction of 14.97% in pesticide expenditure. Moreover, the reduction effect of AMS on insecticide expenditure is higher than that on herbicide expenditure. (b) The reduction effect of AMS on pesticide input is much greater in plain areas than in mountainous and hilly areas, with reductions of 74.65% and 44.25%, respectively. (c) The average pesticide input of large-scale farmers is much lower than that of small-scale farmers. However, the reduction effect of AMS on pesticide input is greater for small-scale farmers than for large-scale farmers, with reductions of 42.464 yuan/mu and 133.122 yuan/mu, respectively.

Based on the above findings, the following policy implications can be drawn: (a) Given the significant negative impact of AMS on pesticide input, it is essential to continue promoting the development of AMS. Policy makers should consider the significant factors affecting farmers' adoption of AMS, such as education level, household income, agricultural subsidies, ownership of agricultural mechanization, hiring laborers, and purchases of agricultural inputs through e-commerce platforms. Policies such as technical training, financial subsidies, increased supply of AMS, and promotion of information dissemination on AMS should be implemented to promote the development of AMS. (b) Policymakers should increase the supply and promotion of AMS in mountainous and hilly areas, and improve the adoption rate of AMS in these regions. (c) Effectively integrating the cropland resources of small-scale farmers and expanding their farming operations can help improve the reduction effect of AMS on pesticide input.

## Data availability statement

The original contributions presented in the study are included in the article/supplementary material, further inquiries can be directed to the corresponding author.

## Author contributions

Conceptualization, data, and writing—original draft preparation: XL. Writing—review and editing: MZ. All authors have read and agreed to the published version of the manuscript.
